# Supplementary material for: HIVprotI: an integrated web based platform for prediction and design of HIV proteins inhibitors
Source: J Cheminform. 2018 Mar 9;10:12. doi: 10.1186/s13321-018-0266-y (PMC5845081; doi:10.1186/s13321-018-0266-y)
Supplement: Supplementary file 2 — Additional file 2. Source code of HIVProtI web server. [file 13321_2018_266_MOESM2_ESM.zip › HIVprotI_Source-code/hivprotI/weka-3-6-6/doc/weka/gui/beans/ChartEvent.html]

ChartEvent


---


|  |  |  |
| --- | --- | --- |
| **Overview** \| **Package** \| **Class** \| **Tree** \| **Deprecated** \| **Index** \| **Help** \| **Weka's home** | |  |
| **PREV CLASS**   **NEXT CLASS** | **FRAMES**    **NO FRAMES**     **All Classes** |  |
| SUMMARY: NESTED \| FIELD \| CONSTR \| METHOD | DETAIL: FIELD \| CONSTR \| METHOD |  |


---


## weka.gui.beans Class ChartEvent

```
java.lang.Object
  java.util.EventObject
      weka.gui.beans.ChartEvent
```

**All Implemented Interfaces:**: java.io.Serializable

---

``` public class ChartEvent extends java.util.EventObject ```

Event encapsulating info for plotting a data point on the StripChart

**Version:**
:   $Revision: 1.4 $

**Author:**
:   Mark Hall

**See Also:**: Serialized Form

---

**Constructor Summary**

`ChartEvent(java.lang.Object source)`
  
          Creates a new `ChartEvent` instance.

`ChartEvent(java.lang.Object source,
java.util.Vector legendText,
double min,
double max,
double[] dataPoint,
boolean reset)`
  
          Creates a new `ChartEvent` instance.


| **Method Summary** | |
| --- | --- |
| `double[]` | `getDataPoint()`             Get the data point |
| `java.util.Vector` | `getLegendText()`             Get the legend text vector |
| `double` | `getMax()`             Get the max y value |
| `double` | `getMin()`             Get the min y value |
| `boolean` | `getReset()`             get the value of the reset flag |
| `void` | `setDataPoint(double[] dp)`             Set the data point |
| `void` | `setLegendText(java.util.Vector lt)`             Set the legend text vector |
| `void` | `setMax(double m)`             Set the max y value |
| `void` | `setMin(double m)`             Set the min y value |
| `void` | `setReset(boolean reset)`             Set the reset flag |

**Methods inherited from class java.util.EventObject**

`getSource, toString`

**Methods inherited from class java.lang.Object**

`equals, getClass, hashCode, notify, notifyAll, wait, wait, wait`

**Constructor Detail**

### ChartEvent

```
public ChartEvent(java.lang.Object source,
                  java.util.Vector legendText,
                  double min,
                  double max,
                  double[] dataPoint,
                  boolean reset)
```

:   Creates a new `ChartEvent` instance.

    **Parameters:**: `source` - the source of the event: `legendText` - a vector of strings to display in the legend: `min` - minimum y value: `max` - maximum y value: `dataPoint` - an array of y values to plot: `reset` - true if display is to be reset

---


### ChartEvent

```
public ChartEvent(java.lang.Object source)
```

:   Creates a new `ChartEvent` instance.

    **Parameters:**: `source` - the source of the event


**Method Detail**

### getLegendText

```
public java.util.Vector getLegendText()
```

:   Get the legend text vector

    :   **Returns:**: a `Vector` value

---


### setLegendText

```
public void setLegendText(java.util.Vector lt)
```

:   Set the legend text vector

    :   **Parameters:**: `lt` - a `Vector` value

---


### getMin

```
public double getMin()
```

:   Get the min y value

    :   **Returns:**: a `double` value

---


### setMin

```
public void setMin(double m)
```

:   Set the min y value

    :   **Parameters:**: `m` - a `double` value

---


### getMax

```
public double getMax()
```

:   Get the max y value

    :   **Returns:**: a `double` value

---


### setMax

```
public void setMax(double m)
```

:   Set the max y value

    :   **Parameters:**: `m` - a `double` value

---


### getDataPoint

```
public double[] getDataPoint()
```

:   Get the data point

    :   **Returns:**: a `double[]` value

---


### setDataPoint

```
public void setDataPoint(double[] dp)
```

:   Set the data point

    :   **Parameters:**: `dp` - a `double[]` value

---


### setReset

```
public void setReset(boolean reset)
```

:   Set the reset flag

    :   **Parameters:**: `reset` - a `boolean` value

---


### getReset

```
public boolean getReset()
```

:   get the value of the reset flag

    :   **Returns:**: a `boolean` value


---


|  |  |  |
| --- | --- | --- |
| **Overview** \| **Package** \| **Class** \| **Tree** \| **Deprecated** \| **Index** \| **Help** \| **Weka's home** | |  |
| **PREV CLASS**   **NEXT CLASS** | **FRAMES**    **NO FRAMES**     **All Classes** |  |
| SUMMARY: NESTED \| FIELD \| CONSTR \| METHOD | DETAIL: FIELD \| CONSTR \| METHOD |  |


---
